# Paroxysmal Sympathetic Hyperactivity after Cardiac Arrest in a Young Male

**DOI:** 10.7759/cureus.3028

**Published:** 2018-07-22

**Authors:** Tanveer Singh, Tanureet K Arora, Prabhjot Bedi, Sanjana Kashinath

**Affiliations:** 1 Hospital Medicine, Rochester General Hospital, Rochester, USA; 2 Internal Medicine, Rochester General Hospital, Rochester, USA; 3 Hospital Medicine, UPMC East, Pittsburgh, USA

**Keywords:** autonomic nervous system diseases, paroxysmal sympathetic hyperactivity, cardiac arrest, dysautonomia, brain injury, traumatic brain injury, paroxysmal autonomic instability with dystonia

## Abstract

Paroxysmal sympathetic hyperactivity (PSH) is a syndrome of an increased sympathetic drive after brain injury. PSH has been previously referred with multiple different names. It is seen most commonly after a traumatic brain injury, but rarely it has been reported after infections, brain malignancies, and brain injury after cardiac arrest. We present a case of a young male who developed PSH after cardiac arrest and will discuss clinical features and various management options.

## Introduction

Paroxysmal sympathetic hyperactivity (PSH) is a disorder of autonomic nervous system. The site of impairment in the brain has not been identified so far. PSH is seen in intensive care unit (ICU), rehabilitation wards, and long-term care (LTC) hospitals. The conceptual definition of PSH was framed by a 26-member working group and it was published in 2014. It defines PSH as “A syndrome recognized in a sub-group of survivors of severe acquired brain injury, of simultaneous, paroxysmal transient increase in sympathetic (elevated heart rate, blood pressure, respiratory rate, temperature, sweating) and motor (posturing) activity” [1]. It can mimic multiple disorders and it needs appropriate workup as described in our case.

## Case presentation

A 30-year-old male with no past medical history moved from Puerto Rico three weeks prior to admission. He was found unresponsive at home with foamy secretions around his mouth. Paramedics found him apneic and pulseless. Cardiopulmonary resuscitation (CPR) was initiated and he had the return of spontaneous circulation after prolonged CPR. He was admitted to ICU and started on therapeutic hypothermia. His urine toxicology revealed cocaine, benzodiazepines and cannabinoids and most likely etiology for his cardiac arrest was thought to be due to overdose. Antibiotics (vancomycin and piperacillin-tazobactam) and vasopressors were initiated for septic shock along with mechanical ventilation and intubation for respiratory failure. His significant laboratory studies were as below in Table 1.

**Table 1:** Laboratory studies. AST: Aspartate aminotransferase; ALT: Alanine aminotransferase.

Laboratory studies	Result (normal range)
Leukocyte count	23,000/µL (4.0-11.0)
Creatinine	2.3 mg/dL (0.5-0.9)
Serum Bicarbonate	18 mEq/L (20-31)
AST	1280 units/L (7-37)
ALT	1570 units/L (10-49)
Lactic acid	13.8 mmol/L (0.4-2.0)

**Figure 1:**
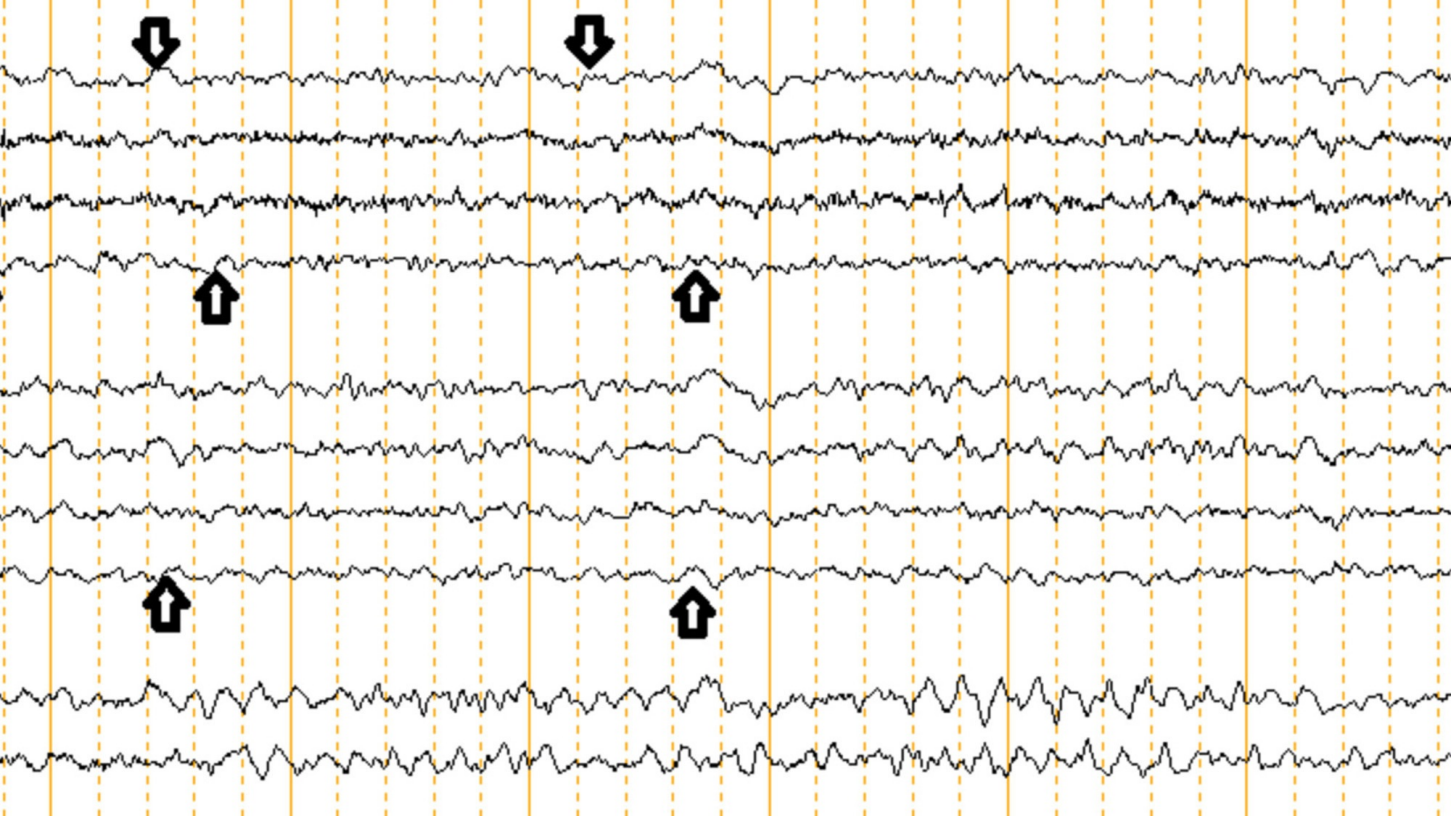
EEG showing diffuse slowing indicating generalized encephalopathy. EEG: Electroencephalogram

He remained unresponsive on discontinuation of propofol but he showed muscle twitching. Electroencephalogram (EEG) showed encephalopathy without seizure activity (Figure 1).

**Figure 2:**
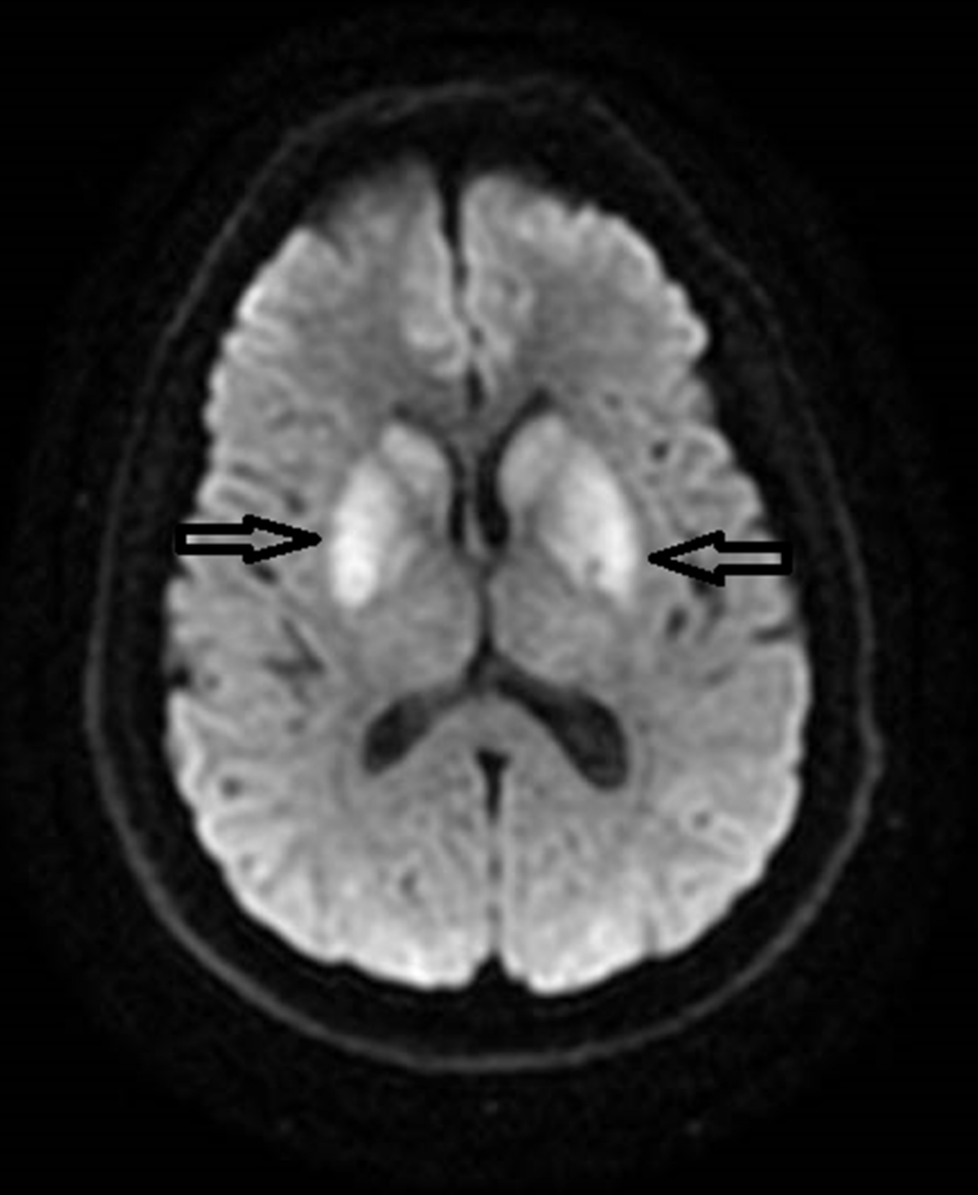
MRI brain axial DWI showing restricted diffusion in bilateral basal ganglia. MRI: Magnetic resonance imaging; DWI: Diffusion weighted imaging.

**Figure 3:**
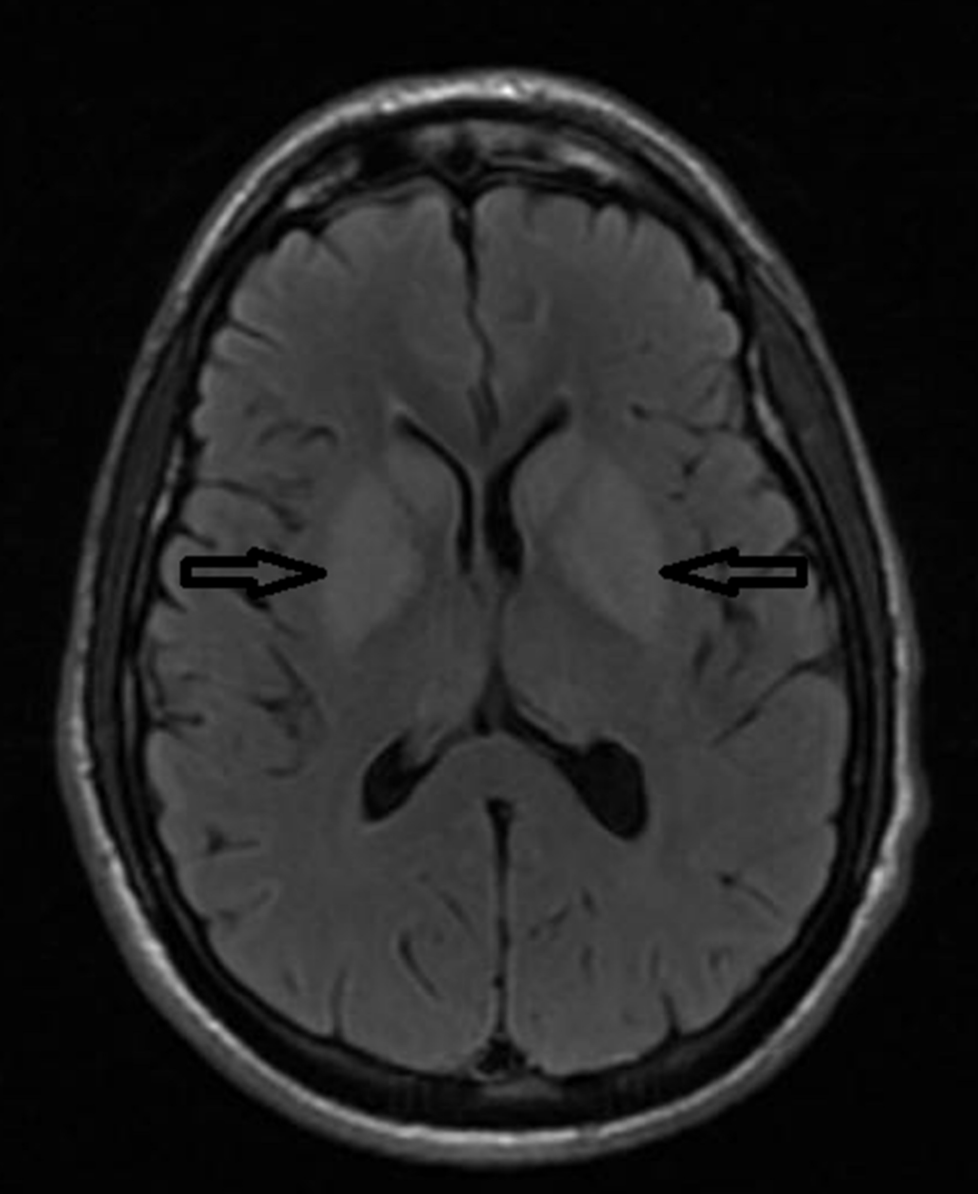
MRI brain axial T2 FLAIR sequence showing hyperintensities in bilateral basal ganglia. MRI: Magnetic resonance imaging; FLAIR: Fluid attenuated inversion recovery.

On day three he had repeated episodes of twitching, decerebrate posturing, eyes rolling for which propofol was restarted. Repeat EEG showed similar results to prior EEG. Magnetic resonance imaging (MRI) brain showed diffuse injury and restricted diffusion involving both basal ganglia, bilateral frontal and occipital cortices (Figures 2, 3).

He continued to have repeated episodes of decerebrate posturing, rhythmic jaw movements, sweating, fever, and tachycardia. He ultimately required tracheostomy and gastrostomy tube placement. After initial antibiotic treatment of one week, his sputum cultures grew *Pseudomonas aeruginosa* which resolved with a course of gentamicin nebulizers.

Infectious disease was consulted for repeated fevers. He had negative blood cultures, sputum cultures, and urine *Legionella *antigen. His urinalysis was negative for infection and no diarrhea, decubitus ulcers or rash was identified. Thyroid function tests (TFT) did not show hyperthyroidism, and ultrasound (US) of the abdomen was negative for acalculous cholecystitis. Hepatitis B and hepatitis C serologies, interferon-gamma release assay, and human immunodeficiency virus (HIV) were negative. He had constant lip smacking resulting in gum bleeds, but no evidence of oral infection was noticed. Tagged white blood cell scan showed uptake in the region of cecum and ascending colon but colonoscopy did not show any evidence of inflammatory bowel disease or colitis.

While getting this workup he was started on acetaminophen for fever and baclofen for rigidity. At this point in time, he was diagnosed with PSH as the workup described above was negative. He was initially started on the regimen of bromocriptine and propranolol but his response was poor. Clonidine and diazepam were added to the regimen which eventually helped with his symptoms. He was then discharged to an LTC facility after a prolonged hospital course. Currently, he has improved and is slightly responsive. He was able to say a few words to his family and identify his family members, but continues to be bed bound.

## Discussion

PSH is characterized by increased sympathetic drive after severe brain injury. It has been labeled variously in literature but some commonly used names are autonomic dysfunction syndrome [2], sympathetic storming [3], acute midbrain syndrome [4], diencephalic seizures [5], and hyperpyrexia associated with muscle contraction [6]. We have used the term paroxysmal sympathetic hyperactivity as described by the consensus working group [1].

In our patient who had a cardiac arrest and a prolonged downtime, brain hypoxia was the causative factor for PSH. Similar cases have been seen in patients with traumatic brain injury (TBI), hydrocephalus, brain tumors, cerebrovascular accident, and subarachnoid hemorrhage [7]. There have been case reports of PSH caused by pneumococcal meningitis [8] and tuberculous meningitis [9,10]. Like our patient, cardiac arrest and eventual hypoxic brain injury causing PSH has also been described [11]. Pre-admission hypoxia was reported in 62% and 29% of cases in two case series involving adult [12] and pediatric [13] patients, respectively.

Clinical features of our patient were fever, tachycardia, high blood pressure, diaphoresis, decorticate posturing, pupillary dilatation, and teeth grinding due to rhythmic jaw movements. Other features also reported in the literature are agitation, and decerebrate posturing [14]. The symptoms of PSH are paroxysmal in nature and generally last for a few hours. Their onset can be anytime between the first week of presentation and up to six months of presentation. The symptoms can also last for more than six months. When it initially develops PSH can mimic multiple disorders, most important among them are sepsis, serotonin syndrome, malignant hyperthermia, pheochromocytoma, salicylate poisoning, cocaine or amphetamine abuse, and delirium tremens [14].

PSH can be seen in a hospitalized patient after multiple insults. It is worthwhile to rule out all the infectious etiologies of fever. Basic imaging including chest radiograph (especially patients who are on a ventilator) should be performed. Blood and sputum should be cultured as the patients are at high risk of aspiration pneumonia. HIV screening, hepatitis B, and C screening should be done in patients with high-risk behaviors. US abdomen, liver function tests should be checked to rule-out acalculous cholecystitis. Constant teeth grinding in some patients and oral injury can lead to oral abscesses and dental exam should be a part of the workup. There can be certain vasculitides and inflammatory joint diseases (IJD) that can present with fever and elevated inflammatory markers but most of these disorders have specific diagnostic criteria and/or specific symptoms. A certain level of suspicion is required before working up these disorders. Sometimes a patient’s inability to give any history and unknown past medical history can be a barrier, so it is not unreasonable to work up for vasculitides and IJD if the clinical suspicion is there.

MRI brain with diffusion-weighted imaging plays an important role to rule out other etiologies [15]. EEG is an integral part of workup to check for seizure activity. Workup for non-infectious etiologies includes TFT, urine metanephrines, complete medication review, salicylate level and urine toxicology.

PSH is an uncommon entity with unclear pathophysiology which is managed symptomatically with medications from different pharmacologic classes. As PSH is generally evident when patient’s sedation is reduced and the patient is coming off the ventilator, giving sedation back to the patient with propofol, dexmedetomidine, fentanyl, morphine, and benzodiazepines is generally useful in an acute situation but it can delay the extubation. These medications should be limited for acute attacks only rather than continuous management [16].

A patient should always be on some scheduled medication regimen to prevent acute attacks. We used clonidine and propranolol for our patient. There is plenty of evidence showing the usefulness of propranolol [6,12,14]. Its non-selective action on both alpha and beta receptors helps in controlling symptoms like tachycardia, hypertension, and diaphoresis. It even helps in agitation control and hyperthermia. Other beta blockers like labetalol have also been tried successfully in a case report [17].

Clonidine is a presynaptic alpha 2 agonist and it is a well-known antihypertensive agent in resistant hypertension. Also, clonidine has some sedating properties. Addition of clonidine to propranolol helps to suppress sympathetic drive even further and better outcomes are seen [12,14]. Clonidine is available both in oral form and as a patch which makes its use even more practical. Dexmedetomidine, another presynaptic alpha 2 agonist, has been shown to be effective and in some reports even more effective than propofol in acute attacks [18]. Tang et al. showed that dexmedetomidine can prevent the occurrence of PSH after TBI [19].

Gabapentin, an antiepileptic drug and commonly used for neuropathic pain, has also shown benefits in PSH. This could be due to its sedating properties [16]. Baclofen is a gamma-aminobutyric acid receptor agonist. It helps with spasticity, rigidity, and pain in patients with PSH thus preventing contractures [20].

## Conclusions

PSH is a widely seen disorder and outcomes are generally poor. Early recognition of this disorder and early treatment may bring positive results. Workup for disorders that mimic PSH should be part of the management plan. There are multiple medications available for use in a patient presenting with PSH. There are no guidelines available for an appropriate regimen and most of the patients require multiple medications from different classes for symptom control.
